# Partnership among hospitals to reduce healthcare associated infections: a quasi-experimental study in Brazilian ICUs

**DOI:** 10.1186/s12879-021-05896-0

**Published:** 2021-02-25

**Authors:** Ladjane Santos Wolmer de Melo, Maria Verônica Monteiro de Abreu, Bernuarda Roberta de Oliveira Santos, Maria das Graças Washington Casimiro Carreteiro, Maria Fernanda Aparecida Moura de Souza, Maria Carolina Andrade Lins de Albuquerque, Claudia Fernanda de Lacerda Vidal, Heloisa Ramos Lacerda

**Affiliations:** 1 Clinics Hospital of Pernambuco, Federal University of Pernambuco, Recife, Pernambuco Brazil; 2 Oswaldo Cruz Hospital, University of Pernambuco, Recife, Pernambuco Brazil; 3 PROCAPE Hospital, University of Pernambuco, Recife, Pernambuco Brazil; 4 Pelopidas Silveira Metropolitan Hospital, Recife, Pernambuco Brazil; 5 Getulio Vargas Hospital, Recife, Pernambuco Brazil; 6 Department of Clinical Medicine, Federal University of Pernambuco, Recife, Pernambuco Brazil

**Keywords:** Healthcare-associated infections, Breakthrough series, Collaborative, Intensive care units, Prevention bundles

## Abstract

**Background:**

Healthcare-associated infections (HAIs) are relevant in developing countries where frequencies can be at least 3 times higher than in developed countries. The purpose of this research was to describe the intervention implemented in intensive care units (ICUs) to reduce HAIs through collaborative project and analyze the variation over 18 months in the incidence density (ID) of the three main HAIs: ventilator associated pneumonia (VAP), central line-associated bloodstream infections (CLABSIs) and catheter-related urinary tract infections (CAUTIs) and also the length of stay and mortality in these ICUs.

**Methods:**

A quasi-experimental study in five public adult clinical-surgical ICUs, to reduce HAIs, through interventions using the BTS-IHI “Improvement Model”, during 18 months. In the project, promoted by the Ministry of Health, Brazilian philanthropic hospitals certified for excellence (HE), those mostly private, certified as excellence and exempt from security contributions*,* regularly trained and monitored public hospitals in diagnostics, data collection and in developing cycles to improve quality and to prevent HAIs (bundles). In the analysis regarding the length of stay, mortality, the IDs of VAP, CLABSIs and CAUTIs over time, a Generalized Estimating Equation (GEE) model was applied for continuous variables, using the constant correlation (exchangeable) between assessments over time. The model estimated the average difference (β coefficient of the model) of the measures analyzed during two periods: a period in the year 2017 (prior to implementing the project) and in the years 2018 and 2019 (during the project).

**Result:**

A mean monthly reduction of 0.427 in VAP ID (*p* = 0.002) with 33.8% decrease at the end of the period and 0.351 in CAUTI ID (*p* = 0.009) with 45% final decrease. The mean monthly reduction of 0.252 for CLABSIs was not significant (*p* = 0.068). Length of stay and mortality rates had no significant variation.

**Conclusions:**

Given the success in reducing VAP and CAUTIs in a few months of interventions, the achievement of the collaborative project is evident. This partnership among public hospitals/HE may be applied to other ICUs including countries with fewer resources.

**Supplementary Information:**

The online version contains supplementary material available at 10.1186/s12879-021-05896-0.

## Background

Healthcare-associated infections (HAIs) are relevant to global public health, especially in developing countries where the frequency may be at least 3 times higher than in developed countries [1]. In Brazil, the incidence density (ID) of HAIs related to devices in the year 2016 indicated ventilator associated pneumonia (VAP) of 13.6/1000 days, central line-associated bloodstream infections (CLABSIs) of 4.6/1000 days and catheter-related urinary tract infections (CAUTIs) of 5.1/1000 days [2], while the 2016 European annual report registered IDs for VAP of 3.9/1000 days, CLABSIs of 1.7/1000 days and CAUTIs of 2.1/1000 days [3]. Although these are frequent adverse events, with high morbidity and mortality rates and high costs, HAIs are recognized as being preventable in up to 70% of cases [4].

Outstanding amongst the strategies for healthcare quality improvement, including the reduction of HAIs, is the Breakthrough Series Collaborative method - BTS, by the Institute for Healthcare Improvement - IHI [5]. Since 1996, this has been implemented in a number of health systems, initially and chiefly in developed economies in North America, Europe and Australia [6], and even in low-to-middle income countries, such as those in Latin America and Africa [7].

In 2018, Susan Wells et al. published the results of a thorough systematic review, studying collaborative methods published between 1995 and 2014 and concluded that, despite methodological limitations and little description regarding aspects of implementation, they were nonetheless effective in improving processes and results. In 83% of hospital studies, there was an improvement in at least one of the investigated indicators and, when a more conservative criterion was used, this effectiveness was 73% [8].

In Brazil, where there are interstate and regional socioeconomic differences, quality improvement programs (QIPs) using the BTS collaborative method are still rare, although they were employed in 2015/16 in the southeast of the country for safe childbirth [9] and in intensive care units (ICUs) in Midwest, Southeast and South Brazil in combating HAIs [10], with successful results. However, no publications on QIPs were observed in the current literature in ICUs in the Northeastern region of the country, which has more limited resources [11].

In order to reduce the incidence of HAIs by 30% in 18 months the Brazilian Ministry of Health promoted the collaborative project “The large-scale improvement of patient safety in Brazil”, through the Institutional Development Program of the Integrated Health System (PROADI-SUS), with BTS large-scale improvement methodology. In this program, Brazilian philanthropic hospitals of excellence (HE) applied their technical capacity and knowledge to promote healthcare improvement in public hospitals across the country (Unified Health System, treated in Brazil by its Portuguese acronym SUS) [1].

The project took place in 119 adult Brazilian intensive care units (ICUs), including about 1200 beds in total. Each of the five Brazilian HE guides 24 institutions in 25 out of 26 existing states and the Federal District throughout the country [12]. Five of which were in the Metropolitan Region of Recife, in the northeast of the country, with a population of around 4 million.

The purpose of this research was to describe how the PROADI-SUS project was implemented in these five ICUs, and to assess whether the 30% reduction over 18 months goal was achieved, in the incidence density (ID) of the three main HAIs: VAP, CLABSIs and CAUTIs [13], as well as the length of stay and mortality in these ICUs. It should be noted that the majority of existing collaborative projects focus on indicators for only one or two HAIs [8].

## Methods

### Study location

This study was conducted in 48 adult ICU beds in five public tertiary hospitals in Recife, in the Northeastern region of Brazil, from January/2018 to June/2019. These were clinical-surgical ICUs with an admission rate of around 1800 patients per year.

### Study setting and design

In this quasi-experimental time-series study, interventions were carried and data was collected on a monthly basis for 18 months, including all patients admitted to the ICUs. The methodology was the BTS [5] using the “Improvement model”.

Hospital teams were trained by Brazilian philanthropic hospitals certified for excellence (HE) in diagnostics, data collection and in developing cycles to improve quality and to prevent HAIs (VAP, CLABSIs and CAUTIs). In Brazil, according to Decree 8242 of 04/23/2014, HE are those certified for excellence and exempt from social security contributions, as long as part of their services be offered to SUS (Unified Health System, treated in Brazil by its Portuguese acronym SUS). These institutions are mostly private, offering assistance, teaching, and research activities, to qualify the public health system in exchange for the non-payment of taxes that should be collected [14]. These face-to-face and online training sessions took place during periodic sessions for sharing questions, experiences and results. The hospitals received educational visits every 4 months together with online consultations with facilitators on the improvement model, patient safety, intensive care and infectious diseases.

The methodology at the original project and at this study included following instructional diagrams demonstrating the preventive measures for HAIs, implemented through PDSA (Plan-Do-Study-Act) rapid cycle testing [5]. PDSAs are improvement tests when changes were first performed with a small group of patients and healthcare professionals, thereby enabling small-scale testing to result in learning and adaptations. Once the process was considered suitable for the local reality and the tests had achieved success, it was progressively implemented throughout the rest of the unit. The implemented improvements were monitored by indicators and the institutions received technical visits from the HE.

After each learning session with specialists in quality improvement and HAIs, with the presence of four representatives from each of the hospitals (local management team), periods of action were initiated, during which the teams returned to their organizations and tested the changes in their contexts.

Result indicators were monitored monthly: incidence densities (ID) of the HAIs, length of stay and mortality in ICUs and process indicators: the rate at which devices were used and adherence to the preventive measures (bundles).

The local teams were instructed to carry out systematic educational observations on the diagnoses and adherence to the bundles, with at least 20 monthly observations per indicator, in order to plan new PDSAs. The established bundles were: 1- VAP: oral hygiene, raised headboard (30–45°), reduced sedation, verifying the possibility of extubation, maintaining the cuff pressure of the tracheal cannula (25-30 cm of H_2_O or 20–22 mmHg) and adequate maintenance of the mechanical ventilation system. 2- CLABSIs: on insertion of the central venous catheter (CVC) – check indications, precautions for maximum barrier, skin antisepsis with chlorhexidine, optimal selection of insertion site, adequate dressing after insertion; maintenance of CVC - indication of permanence, aseptic technique in handling, maintenance of the infusion system, correct dressing technique. 3- CAUTIs: when inserting the urinary catheter (UC) – check indication, aseptic technique; maintenance of the UC - permanence of the closed system, correct technique during drainage manipulation, hygiene of the urethral meatus, check the need to maintain the UC.

The local teams monitored and shared the active PDSAs with the ICU team, on a weekly basis - through rounds -, and the indicators, on a monthly basis. The monthly data on the frequency of HAIs and adherence to bundles were recorded on a digital platform to be analyzed in order to direct the necessary actions to improve the team’s performance.

The aggregate results of the 119 hospitals participating in the Collaborative until April 2019 have shown reductions of 41% in CLABSI, 48% in CAUTI and 28% of VAP [12].

### Definitions

Surveillance of the HAIs was conducted by professionals trained in infection control, using the definitions of the US Centers for Disease Control and Prevention – CDC [15] and their incidence was expressed as cases per 1000 devices-day, obtained by the ratio of the monthly number of cases of infection by the number of patients using the device-day related to this infection.

The utilization rate of the devices was the percentage calculated by adding the number of patients using the device-day divided by the sum of the total number of patient-days in the same period.

The percentage of adherence to bundles was assessed by dividing the number of patients observed with 100% adherence to all items in the bundle by the number of patients observed with the device.

### Microbiological methods

All isolates were identified by manual or automated methods and confirmed with the Vitek 2 system (bioMerieux Vitek, Inc., Hazelwood, MO).

### Ethical aspects

This research was promoted and authorized by Brazilian Ministry of Health, carried out through the Institutional Development Program of the Integrated Health System PROADI-SUS [16] and approved by the Ethics Committee of the Hospital das Clínicas - UFPE, under No. 3,307,293.

### Statistical analysis

In the presentation of hospital characteristics, absolute and percentage frequency measurements were performed for categorical variables, and the mean and standard deviation were calculated, as well as the medians and interquartile ranges for quantitative variables. The hypothesis of normality for incidence densities (ID) was tested by the Shapiro-Wilks test, and the hypothesis of normality was accepted.

In the analysis regarding the length of stay, mortality, the IDs of VAP, CLABSIs and CAUTIs over time, Generalized Estimating Equation (GEE) model was applied for continuous variables, using the constant correlation (exchangeable) between assessments over time. The model estimated the average difference (β coefficient of the model) of the measures analyzed during two periods: a period in the year 2017 (prior to implementing the project) and in the years 2018 and 2019 (during the project).

The Spearman’s correlation coefficient was estimated in the assessment of process indicators as explanatory variables of the behavior of the result indicators. The percentage of variation in the intervention period was based on the difference between the result indicator in January 2018 and June 2019. All tests of statistical significance were bilateral, with a significance level of 0.05 (*p* < 0.05). All data analyzes were performed using STATA 14.

## Results

### Characteristics of the hospitals in the study

Five ICUs were selected by the participating hospital team totaling 48 beds in the five hospitals included in the study. Each hospital could only choose one unit to participate in the project. Three were general ICUs, one was a cardiac ICU and the other was a neurological ICU. Table 1 presents the characteristics of the studied hospitals, in which the mean number of patients-day admitted to the analyzed ICUs varied between 179 and 298 patients each month and was higher in hospitals H1, H2 and H3 when compared to hospitals H4 and H5, which presented a lower mean patient-day rate. The mean number of hospitalizations per month ranged from 23 to 31 patients. In the half-yearly assessment, there was an increase in the implementation of PDSAs in most hospitals, with hospitals H1, H2 and H5 presenting a higher percentage of total implementation when compared to the others (Table 1).

**Table 1:** Characteristics of the five hospitals assessed from January 2018 to June 2019

Characteristics	H1	H2	H3	H4	H5
**Total number of beds**	234	444	170	415	413
**Number of beds in ICU**	30	31	30	17	12
**Number of beds studied**	10	10	10	10	8
**Type of ICU studied**	Cardiology	General	Neurology	General	General
**Patient-day ICU admissions per month**^**ab**^	291 ± 22 (216–306)	298 ± 11 (270–310)	297 ± 19 (210–310)	191 ± 32 (101–246)	179 ± 22 (116–219)
**Monthly number of ICU admissions**^**a**^	31 ± 8 (12–45)	23 ± 7 (12–45)	30 ± 8 (15–51)	25 ± 5 (13–33)	24 ± 6 (8–33)
**PDSAs implemented/carried out (% implemented)**					
1° semester	21/36 (58.3%)	9/11 (81.8%)	3/49 (6.1%)	4/30 (13.3%)	19/30 (63.3%)
2° semester	13/18 (72.2%)	2/4 (50.0%)	2/5 (40.0%)	11/25 (44.0%)	14/16 (87.5%)
3° semester	8/9 (88.9%)	2/3 (66.7%)	12/13 (92.3%)	2/3 (66.7%)	4/5 (80.0%)
**Total**^**d**^	**42/63 (66.7%)**	**13/18 (72.2%)**	**17/67 (25.4%)**	**17/58 (29.3%)**	**37/51 (72.5%)**
**Number of monthly meetings held during the period**^**c**^	4 (3–4)	1 (0.25–2.75)	2 (1–3)	1 (1–3)	3 (3–3.75)
**Percentage of patients with daily defined objectives in ICU**^**c**^	100 (100–100)	Not assessed	31.1 (13.6–66.9)	53.8 (50.8–56.9)	100 (100–100)
Assessment period of the defined objectives	Jan/19 to June/19	–	Jan/19 to June/19	Nov/17 to July/18	Jan/18 to June/19
**Percentage of patients who received daily multidisciplinary visits in ICU**	100 (100–100)	19.9 (19.1–22.1)	23.1 (13.5–26.9)	61.5 (49.7–64.0)	100 (100–100)
Assessment period of the multidisciplinary visits	Jan/19 to June/19	Feb/19 to June/19	Jun/18 to June/19	Nov/18 to June/19	Jan/18 to June/19

^a^Mean ± SD (min – max) in the studied ICUs

^b^ANOVA: *p* < 0,001 – significant statistical difference: H4 & H5 ≠ H1, H2 & H3

^c^Median (P25 – P75)

^d^There was a significant statistical difference of the H3 & H4 hospitals when compared to the others (*p* < 0.05)

### Result indicators

**Fig. 1:**
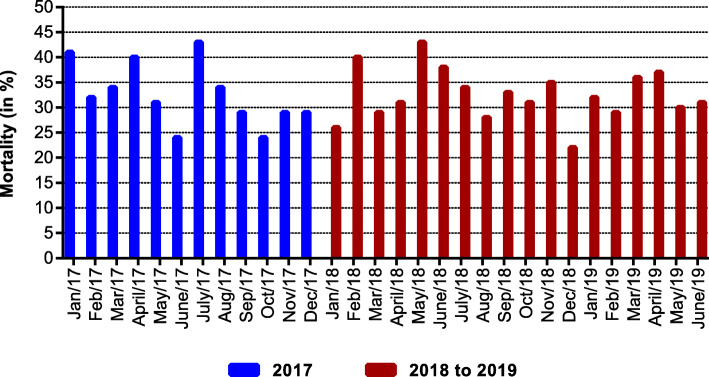
Mortality in ICUs of the five hospitals assessed between January 2017 and June 2019. Before intervention 2017: β = − 0.889 (*p* = 0.089) During intervention 2018 to 2019: β = − 0113 (*p* = 0.646)

With regard to the group of the 5 ICUs studied, over the 18-month period, there was no variation in relation to mortality (2017: β = − 0.889 (*p* = 0.089) and 2018/2019: β = − 0.113 (*p* = 0.646)) (Fig. 1). The mean monthly time of stay decreased in 2017 (β = − 0.292 (*p* = 0.033)) and 2018 (β = − 0.276 (*p* = 0.047)) although in the 6 months of 2019 there was an increase, with no statistical significance (β = 1.399 (*p* = 0.183)). During the intervention, there was a mean monthly reduction of 0.427 in the VAP ID (*p* = 0.002) with a 33.8% decrease at the end of the period, and 0.351 in the CAUTI ID (*p* = 0.009), which corresponded to a 45% decrease at the end. There was a mean monthly reduction of the CLABSI ID of 0.252, which was not significant (*p* = 0.068) (Table 2).

**Table 2:** Incidence densities of HAIs in the ICUs analyzed before and during intervention

Result indicators	Before intervention 2017	During intervention 2018–2019	***P***-value	Variation %
**Incidence density of VAP**				
**Variation over time**				
**Coefficient β (*p*-value)**	− 0.040 (0.934)	− 0.427 (0.002)	–	↓33.8%
**Assessment by hospital**^**b**^				
H1^a^	−3.784 (0.349)	−0.630 (0.069)	0.123	
H2	−2.143 (0.151)	−0.825 (0.015)	0.019	
H3	0.004 (0.988)	−0.302 (0.287)	0.568	
H4	−0.679 (0.135)	−0.040 (0.862)	0.323	
H5	1.167 (0.070)	−0.337 (0.302)	0.114	
**Incidence density of CAUTI**				
**Variation over time**				
**Coefficient β (*p*-value)**	0.300 (0.292)	−0.351 (0.009)	–	↓45.0%
**Assessment by hospital**^**b**^				
H1^a^	−1.131 (0.530)	− 0.720 (0.012)	0.035	
H2	0.097 (0.688)	−0.859 (< 0.001)	< 0.001	
H3	0.624 (0.252)	−0.479 (0.036)	0.057	
H4	0.191 (0.256)	0.264 (0.377)	0.355	
H5	−0.282 (0.490)	−0.111 (0.304)	0.439	
**Incidence density of CLABSI**				
**Variation over time**^**b**^				
**Coefficient β (*p*-value)**	0.251 (0.333)	−0.252 (0.068)	–	NS
**Assessment by hospital**^**b**^				
H1^a^	2.611 (0.016)	0.059 (0.841)	0.055	
H2^a^	0.296 (0.275)	−0.202 (0.152)	0.198	
H3	−0.171 (0.745)	0.021 (0.905)	0.942	
H4	0.118 (0.699)	−0.748 (0.022)	0.067	
H5	−0.181 (0.386)	−0.408 (0.308)	0.408	

*NS* No significant variation

^a^Before the intervention the date for the period between July and December 2017 (6 months)

^b^Linear regression model for each period

### Process indicators

#### VAP

The drop in the rate of monthly percentage utilization of mechanical ventilation in the 5 ICUs from 61.2 + 5.5 to 54.5 + 5.1 (*p* = 0.002) demonstrated a correlation with around 50% (r = 0.485, *p* = 0.007) in the final drop of the VAP ID of 33.8%. A low adherence to the preventive measures was recorded (median 48%) and no correlation with the ID (0.079, *p* = 0.487). There was no correlation between the number of monthly meetings held and the VAP ID (Table 3). Description by hospital regarding the utilization rate of mechanical ventilation and adherence to the single prevention package is presented in Supplementary Table 1.

**Table 3:** Analysis of the process indicators in relation to the IDs of the HAIs

Process indicators	Before intervention 2017	During intervention 2018–2019	***P***-value	Correlation coefficient (***p***-value)
**Mechanical ventilation utilization rate**				
Mean ± SD (in days)	61.2 ± 5.5	54.5 ± 5.1	0.002	0.485 (0.007)^c^
**Adherence to preventive measures of VAP (in %)**				
Median (P_25_ – P_75_)	–	48 (12–84)	–	0.079 (0.487)
**Number of monthly meetings**				
Median (P_25_ – P_75_)	–	2.7 (1.8–3.2)	–	0.049 (0.847)
**UC utilization rate**				
Mean ± SD (in days)	60.6 ± 7.9	43.4 ± 6.1	< 0.001	0.374 (0.042)
**Adherence to preventive measures for INSERTION of UC (in %)**				
Median (P_25_ – P_75_)		79 (50–100)	–	− 0.169 (0.138)^d^
**Adherence to preventive measures for MAINTENANCE of UC (in %)**				
Median (P_25_ – P_75_)	–	71 (60–77)	–	0.289 (0.342)
**Number of monthly meetings**				
Median (P_25_ – P_75_)	–	2.7 (1.8–3.2)	–	0.195 (0.437)
**CVC utilization rate**				
Mean ± SD (in days)	81.7 ± 3.1	77.2 ± 2.9	< 0.001	−0.199 (0.291)^b^
**Adherence to preventive measures for INSERTION of CVC (in %)**				
Median (P_25_ – P_75_)	–	63 (53–73)	–	−0.535 (0.041)^b^
**Adherence to preventive measures for MAINTENANCE of CVC (in %)**				
Median (P_25_ – P_75_)	–	53 (47–58)	–	0.005 (0.986)
**Number of monthly meetings**				
Median (P_25_ – P_75_)	–	2.7 (1.8–3.2)	–	0.288 (0.264)

^a^Data collection on adherence to the preventive measures took place from July/2018

^b^Correlation with CLABSI ID

^c^Correlation with VAP ID

^d^Correlation with CAUTI ID

#### CAUTI

There was a reduction in the monthly percentage utilization rate of UCs in the 5 ICUs from 60.6 + 7.9 to 43.4 + 6.1 (*p* = < 0.001) demonstrating a correlation of 37% (*r* = 0.374, *p* = 0.042) with the final reduction in the ID of 45%. Preventive measures for UC insertion and maintenance demonstrated a median adherence of 79 and 71%, respectively, and there was no correlation with the CAUTI ID. There was no correlation between the number of monthly meetings held and the CAUTI ID (Table 3). Description by hospital of the UC utilization rate and the respective adherence to the preventive insertion and maintenance measures is presented in Supplementary Table 2.

#### CLABSI

The monthly percentage utilization rate of the central venous catheter decreased from 81.7 + 3.1 to 77.2 + 2.9 (*p* = < 0.001) but there was no correlation with a reduction in the CLABSI ID. The median adherence to the preventive measures for CVC insertion of 63% correlated with 53% (*r* = − 0.535, *p* = 0.041) of reduction in the ID (not significant). The median adherence to the maintenance measures was 53% and there was no correlation with the ID of the infection. There was no correlation between the number of monthly meetings held and the CLABSI ID (Table 3). Description by hospital of the CVC utilization rate and respective adherence to preventive insertion and maintenance measures is presented in Supplementary Table 1.

## Discussion

Our study has demonstrated that the “Improvement Model” method is effective in implementing projects for healthcare improvement in public hospitals aided by philanthropic hospitals of excellence (HE) [16]. After just a few months of preventive interventions in ICUs, a significant result was obtained in reducing VAP and CAUTIs. Internal infection control programs were already in place in ICUs, however, the incidence of HAIs remained high. Thus, this project, promoted by the Brazilian Ministry of Health, based on the improvement model of the BTS-IHI [5], provided the ICUs with an opportunity to gradually implement or ratify the preventive measures of the bundles, using a new methodology whereby participation by the areas in question (ICUs) gained prominence and empowerment over the infection prevention process [13].

The main result indicators of this quasi-experimental study, in relation to the group of 5 ICUs studied, were an average monthly decrease of 0.427 (*p* = 0.002) in the VAP ID and 0.351 (*p* = 0.009) in the CAUTI DI over a period of 18 months. The final reduction during this period of the VAP ID by 34% and the CAUTI ID by 45%, is in accordance with the prevention percentages obtained in the revised multifaceted interventions, from 2005 to 2016, by Schreiber et al., also including high-income countries. The authors considered that the potential for reducing HAI by around 30 to 50%, through evidence-based strategies, demonstrates that the current recommendations have not been sufficiently implemented [17]. The mean monthly reduction of CLABSI ID by 0.218 (*p* = 0.07) was not significant, although it demonstrated a downward trend and reflects the need for more follow-up time for this measure. It is probable that the moderate adherence to the preventive measures for CLABSIs (63 and 53%), even resulting in a reduction in the monthly percentage of the CVC utilization rate from 81.7 + 3.1 to 77.2 + 2.9 (*p* = < 0.001) was insufficient to reduce the incidence of CLABSIs. Our result coincides with the findings of a large study on implementing the CLABSIs bundle in ICUs in the USA, which demonstrated that CLABSIs are only reduced when adherence to the bundle is at least 95% [18].

The length of stay and mortality throughout the 18 months did not significantly decrease. In part, this may signify that patient death with HAIs is not always only attributed to this adverse event [19] and, since there are multiple causes, the role of infection is not always clear [4]. However, since it is indisputable that HAIs increase mortality and hospital stay [20] we believe that the reductions obtained in the ID were not sufficient to alter the length of stay and mortality in the ICUs.

With regard to the process indicators in the prevention of VAP, a decrease was observed in the rate of the monthly percentage utilization of mechanical ventilation in the 5 ICUs, from 61.2 + 5.5 to 54.5 + 5.1 (*p* = 0.002) which correlates with around 50% (0.485, *p* = 0.007) of the reduction, at the end of the period, in the VAP ID. The importance of the utilization rate and its reduction explain the significant reduction in VAP ID even with insufficient adherence to the bundle [21]. However, it is important to emphasize that the median of 38% adherence to the preventive measures of VAP is not realistic, since there occurred a divergence in the understanding of the cuff pressure measurement methodology, thereby negatively interfering with the assessment of the adherence to the bundle. In summary, the 35% reduction in the VAP ID was associated with a drop in the utilization of mechanical ventilation (*r* = 0.485, *p* = 0.007), and a possible contribution from other preventive measures although registered at low levels.

In processes involving the prevention of CAUTI, there was also a significant decrease in the monthly percentage utilization of UCs in the 5 ICUs, from 60.6 + 7.9 to 43.4 + 6.1 (*p* = < 0.001) representing a 37% correlation with a reduction in the CAUTI ID at the end of the period (0.374, *p* = 0.042). Adherence to the preventive measures for insertion and maintenance of the UC presented a median of 79 and 71%, respectively, and there was no correlation with the CAUTI ID. As Titsworth demonstrated, there is a linear relationship between the UC utilization rate and the CAUTI ID that explains the 45% reduction in the ID [22].

Unlike most intervention projects that aim to reduce just one or two HAIs, [8, 17] our study has demonstrated that it is possible to confront 3 HAIs while at the same time expanding interventions. This aspect is corroborated by the work of Miller et al. [23] when relevant reductions were obtained in ICUs of the 3 HAIs over an intervention period of 2 years and in the same follow-up period. This seeks to improve care and the overall safety culture in the work unit, since it is known that there are many other infections that have been generated by health care, little studied in projects [17], which indirectly may be avoided by improving the patient care.

The data on infections were collected by the hospitals themselves by trained professionals, using the criteria of the CDC [15]. Differences among hospitals in relation to the adherence to the strategies proposed, as well as the number of monthly meetings can be explained by the characteristics of the hospitals in the study (Table 1) and because some diverse aspects, such as: scarce or existing resources, including human resources, willingness and availability of the ICU team management.

The major flaws in executing the project were those related to registering and adherence to bundles, which did not occur in the expected frequencies (Table 3), and which differs from the improvement results of the VAP and CAUTI IDs. Thus, in contrast to the improvement in results, we also observed that some items in the bundles were not executed, there was an insufficient number of audits and that executing professionals failed to complete the medical records. Additionally, the hospitals participating in this research considered that the methodology for measuring the cuff pressure of the orotracheal tube produced measurement inconsistencies, which should have been standard, thereby temporarily affecting the faithful measurement of adherence. All these facts explain the difficulty in assessing the true adherence to bundle items and therefore the registered values (supplementary tables 1 and 2) may not demonstrate the real adherence to the bundles. Certainly, the gaps in execution are related to the results, although the exact preventive contribution of each item in the bundles is unknown [17]. It is our conclusion that with a lower utilization of devices, even with limited adherence to the bundles, there was a significant reduction in infections.

Despite the difficulties in achieving the adherences, we found that the bundles with the best adherence were those for the prevention of CAUTI and the bundles with the worst adherence were those for the prevention of VAP. We believe that this study may be applied to other ICUs as a successful experience within a brief period of time. There are a number of limitations to this study for being quasi-experimental [24], rather than randomized, however, when planning the study, it was considered unethical to maintain a control group without receiving preventive measures.

## Conclusion

Given the success in reducing VAP and CAUTI, there is no doubt regarding the success of the collaborative project, using improvement cycles. The remaining challenges are to guarantee a 95% adherence to the CLABSI prevention bundles, as well as the continued encouragement and involvement of the teams in the processes for consolidating the results. Studies like this one are fundamental for the effective evaluation of the result of the investments of public resources made within this type of financing. This partnership among public hospitals/HE may be applied to other ICUs including countries with fewer resources**.**

## Supplementary Information

**Additional file 1: Supplementary Table 1.** CVC and mechanical ventilation utilization rates and adherence to the preventive measures bundles. **Supplementary Table 2.** UC utilization rate and adherence to the preventive measures bundles.

## Data Availability

The data that support the findings of this study are available from Brazilian Ministry of Health and Institutional Development Program of the Integrated Health System - PROADI-SUS but restrictions apply to the availability of these data, which were used under license for the current study, and so are not publicly available. Data are however available from the authors upon reasonable request and with permission of Brazilian Ministry of Health and Institutional Development Program of the Integrated Health System - PROADI-SUS.

## Abbreviations

BTS: Breakthrough Series Collaborative method
CAUTIs: Catheter-related urinary tract infections
CDC: US Centers for Disease Control and Prevention
CLABSIs: Central line-associated bloodstream infections
CVC: Central venous catheter
GEE: Generalized Estimating Equation
HAIs: Healthcare-associated infections
HE: Brazilian philanthropic hospitals of excellence
ICU: Intensive care units
ID: Incidence density
IHI: Institute of Healthcare Improvement
PDSA: Plan-Do-Study-Act rapid cycle testing
PROADI-SUS: Institutional Development Program of the Integrated Health System
QIPs: Quality improvement programs
SUS: Integrated Health System in Brazil
UC: Urinary catheter
UFPE: Federal University of Pernambuco
VAP: Ventilator associated pneumonia
