# Correction to: Partnership among hospitals to reduce healthcare associated infections: a quasi-experimental study in Brazilian ICUs

**DOI:** 10.1186/s12879-022-07770-z

**Published:** 2022-12-01

**Authors:** Ladjane Santos Wolmer de Melo, Maria Verônica Monteiro de Abreu, Bernuarda Roberta de Oliveira Santos, Maria das Graças Washington Casimiro Carreteiro, Maria Fernanda Aparecida Moura de Souza, Maria Carolina Andrade Lins de Albuquerque, Claudia Fernanda de Lacerda Vidal, Heloisa Ramos Lacerda

**Affiliations:** 1 Clinics Hospital of Pernambuco, Federal University of Pernambuco, Recife, Pernambuco Brazil; 2 Oswaldo Cruz Hospital, University of Pernambuco, Recife, Pernambuco Brazil; 3 PROCAPE Hospital, University of Pernambuco, Recife, Pernambuco Brazil; 4 Pelopidas Silveira Metropolitan Hospital, Recife, Pernambuco Brazil; 5 Getulio Vargas Hospital, Recife, Pernambuco Brazil; 6 Department of Clinical Medicine, Federal University of Pernambuco, Recife, Pernambuco Brazil

## Correction to: BMC Infectious Diseases 21, 212 (2021). https://doi.org/10.1186/s12879-021-05896-0

After publication it was observed that there were data transcription failures, these only occurred in the supplementary file. The errors do not impact the rest of the rest of the paper.

The original article [1] has been updated with the correct supplementary file. Both files (old and new) are available in this correction article.

## Supplementary Information

**Additional file 1**: Incorrect supplementary file as originally published.

**Additional file 2**: Correct supplementary file.
