# Supplementary material for: Correction to: Partnership among hospitals to reduce healthcare associated infections: a quasi-experimental study in Brazilian ICUs
Source: BMC Infect Dis. 2022 Dec 1;22:898. doi: 10.1186/s12879-022-07770-z (PMC9714116; doi:10.1186/s12879-022-07770-z)
Supplement: Supplementary file 1 — Additional file 1: Incorrect supplementary file as originally published. [file 12879_2022_7770_MOESM1_ESM.docx]

**Supplementary Table 1.** CVC and mechanical ventilation utilization rates and adherence to the preventive measures bundles.

| Process indicators | Before intervention  2017 | After intervention  2018 - 2019 | p-value |
| --- | --- | --- | --- |
| CVC utilization rate |  |  |  |
| H1 | 82.6 ± 4.0 | 84.9 ± 7.4 | 0.467 |
| H2 | 92.0 ± 1.9 | 75.4 ± 9.2 | <0.001 |
| H3 | 83.5 ± 6.4 | 77.2 ± 8.5 | 0.038 |
| H4 | 69.9 ± 9.3 | 75.2 ± 9.3 | 0.135 |
| H5 | 84.3 ± 9.7 | 73.1 ± 11.3 | 0.009 |
| Adherence to preventive measures for INSERTION of CVC (in %)^a^ |  |  |  |
| H1 | - | 62 (0 – 80) | - |
| H2 | - | 67 (0 – 83) | - |
| H3 | - | **8**8 **(**7**0 –** 9**6)** | - |
| H4 | - | 76 (67 – 100) | - |
| H5 | - | **87 (**7**1 –** 100**)** | - |
| Adherence to preventive measures for MAINENANCE of CVC (in %) ^a^ |  |  |  |
| H1 | - | 6**5** (48 – 70) | - |
| H2 | - | 32 (0 – 52) | - |
| H3 | - | 89 (71 – 98) | - |
| H4 | - | 52 (38 – 81) | - |
| H5 | - | **66 (43 – 83)** | - |
| Mechanical ventilation utilization rate |  |  |  |
| H1 | 52.1±3.8 | 59.8±7.4 | 0.025 |
| H2 | 68.5±10.3 | 52.8±10.1 | 0.002 |
| H3 | 73.9±7.0 | 60.6±10.1 | <0.001 |
| H4 | 57.5±11.4 | 50.1±8.7 | 0.055 |
| H5 | 53.0±12.1 | 49.0±17.4 | 0.500 |
| Adherence to the VAP prevention bundle (in %) ^a^ |  |  |  |
| H1 | - | 4.5 (0 – 27) | - |
| H2 | - | 18 (0 – 44) | - |
| H3 | - | 82 (75 – 94) | - |
| H4 | - | **51 (42 – 55)** | - |
| H5 | 77 (61 – 86) | 27 (8 – 35) | <0.001 |

^a^ Data collection on adherence to preventive measures took place from June/2018

0

**Supplementary Table 2.** UC utilization rate and adherence to the preventive measures bundles

| Process indicators | Before intervention  2017 | After intervention  2018 - 2019 | p-value |
| --- | --- | --- | --- |
| UC utilization rate |  |  |  |
| H1 | 41.0±6.5 | 38.3±8.9 | 0.494 |
| H2 | 72.2±8.1 | 51.6±8.5 | <0.001 |
| H3 | 54.2±21.8 | 30.7±16.3 | 0.002 |
| H4 | 75.3±10.8 | 60.5±9.4 | <0.001 |
| H5 | 49.0±10.3 | 36.1 ± 10.7 | 0.003 |
| Adherence to the preventive measures for INSERTION of the UC (in %)^a^ |  |  |  |
| H1 | - | **50 (0 – 100)** | - |
| H2 | - | **100 (70 – 100)** | - |
| H3 | - | **100 (100 – 100)** | - |
| H4 | - | **33 (0 – 100)** | - |
| H5 | - | **100 (80 – 100)** | - |
| Adherence to the preventive measures for MAINTENACE of the UC (in %)^a^ |  |  |  |
| H1 | - | 52 (40 – 71) | - |
| H2 | - | **65 (**2**7 – 8**3**)** | - |
| H3 | - | 57 (50 – 96) | - |
| H4 | - | 93 (62 – 96) | - |
| H5 | - | 94 (90 – 100) | - |

^a^ Data collection on adherence to preventive measures took place from June/2018
